# Threshold of long-term survival of a coastal delphinid in anthropogenically degraded environment: Indo-Pacific humpback dolphins in Pearl River Delta

**DOI:** 10.1038/srep42900

**Published:** 2017-02-23

**Authors:** Leszek Karczmarski, Shiang-Lin Huang, Stephen C.Y. Chan

**Affiliations:** 1 The Swire Institute of Marine Science and School of Biological Sciences, Faculty of Science, The University of Hong Kong, Cape d’Aguilar, Shek O, Hong Kong

## Abstract

Defining demographic and ecological threshold of population persistence can assist in informing conservation management. We undertook such analyses for the Indo-Pacific humpback dolphin (*Sousa chinensis*) in the Pearl River Delta (PRD) region, southeast China. We use adult survival estimates for assessments of population status and annual rate of change. Our estimates indicate that, given a stationary population structure and minimal risk scenario, ~2000 individuals (minimum viable population in carrying capacity, MVP_k_) can maintain the population persistence across 40 generations. However, under the current population trend (~2.5% decline/annum), the population is fast approaching its viability threshold and may soon face effects of demographic stochasticity. The population demographic trajectory and the minimum area of critical habitat (MACH) that could prevent stochastic extinction are both highly sensitive to fluctuations in adult survival. For a hypothetical stationary population, MACH should approximate 3000-km^2^. However, this estimate increases four-fold with a 5% increase of adult mortality and exceeds the size of PRD when calculated for the current population status. On the other hand, cumulatively all current MPAs within PRD fail to secure the minimum habitat requirement to accommodate sufficiently viable population size. Our findings indicate that the PRD population is deemed to become extinct unless effective conservation measures can rapidly reverse the current population trend.

In conservation science, a thorough status assessment of a population should ideally consider all five IUCN criteria, including the rate of population change (i.e. decline), distribution range, population structure, population size and risk of extinction^1^. For many cetacean species, however, such a thorough approach is often logistically impossible. Among all five Criteria, the Criterions B and E are particularly challenging, as robust data on distribution and quantitative population viability analysis (PVA) are rare in cetacean studies. This leaves policy makers and conservation managers with a considerable challenge when making management decisions. Incomplete evidence may easily lead to misguided judgments of conservation status, which in turn can further misguide or delay the implementation of appropriate conservation strategies. Such a chain of events may have severe implications for the survival of species and populations^2^,^3^,^4^,^5^.

Under Criterion A of the IUCN Red List Categories and Criteria Version 3.1 1, the population status, either *NT (Near Threatened*), *VU (Vulnerable*), *EN (Endangered*) or *CR (Critically Endangered*), is classified by the percentage of decline within three generations^1^ which comes from relevant trend analyses^6^ and has been applied across variety of species^7^,^8^,^9^,^10^,^11^,^12^. In conservation practice, however, especially when dealing with threatened species and populations, it is important to determine a threshold of population survival; a critical level below which the population and its status begin to decline. Defining a demographic and ecological threshold of population persistence, if timely, can greatly benefit informed conservation management and help prioritizing conservation strategies^13^,^14^,^15^,^16^,^17^.

Population trend is determined by the survival and reproductive rates, as well as the rate of immigration and emigration^15^,^18^,^19^. As most anthropogenic impacts affect population trend by decreasing apparent survival rates^7^,^20^,^21^, we may define a threshold survival rate as the rate below which a negative trend will drive the population into extinction. Such an approach may be particularly useful for many cetacean species as their apparent survival rates can be reliably estimated with photo-ID mark-recapture techniques^7^,^22^. Moreover, since the rate of population decline corresponds to the classification of conservation status under the IUCN Criterion A^1^, we may identify threshold survival rates equivalent of the classification of *NT, VU, EN* or *CR* status.

In the case of small populations, maintaining them at a stationary or slightly increasing trend (*r* ≥ 0) does not necessarily ensure the population long-term persistence because stochastic fluctuations in population numbers tend to decrease the likelihood of survival^13^,^17^,^23^,^24^. As the upper limit of fluctuation is determined by carrying capacity (*K*_0_) of the habitat, for a population to persist, a threshold value of *K*_0_, termed the minimum viable population in carrying capacity (MVP_k_), has to be spared the risk of random extinction caused by stochastic population fluctuation^17^,^23^,^24^. As *K*_0_ corresponds to the area of suitable habitat, a threshold habitat size, the minimum area of critical habitat (MACH) can be further defined to accommodate MVP_k_ of animals^25^,^26^. These estimates, however, have never been applied yet in analyses of cetacean populations.

Indo-Pacific humpback dolphins (*Sousa chinensis*), locally in China and Taiwan known as Chinese White Dolphins, inhabits shallow coastal waters of the eastern Indian Ocean and western Pacific^27^,^28^. Their preferred inshore habitats are often in close proximity to areas of intense human activities, which exposes them to various anthropogenic impacts such as coastal fisheries, urban and industrial developments, pollution and disposal of hazardous materials, alteration of coastlines and various degrees of habitat degradation^29^,^30^,^31^,^32^; all of which have been suggested to lead to population decline^4^,^5^,^29^,^33^,^34^,^35^. At present, the IUCN Red List of Threatened Species lists humpback dolphins as *Near Threatened (NT*)^36^. This classification, however, has been challenged^5^ and, following a recent taxonomic revision of the genus *Sousa*^28^, there are numerous indications that all currently recognised species of humpback dolphins may have long been under far greater threat throughout their range than it was previously recognized^5^,^37^,^38^,^39^.

Recent demographic analyses of humpback dolphins in the coastal ecosystem of the Pearl River Delta (PRD, Fig. 1), China, indicate a population decline (*r* = −0.0249) averaging *ca*. 74% decline within three generations^8^. As the PRD region is one of the fastest growing economic regions in the world^40^, which is accompanied by ever increasing anthropogenic pressures on variety of biota^5^,^31^, the humpback dolphin population inhabiting PRD waters is thought to be among the most anthropogenically impacted populations of small cetaceans anywhere in the world^31^,^41^. It is thus highly probable that their survivorship is substantially lower than the currently recognized species’ global *NT* status would imply.

In Hong Kong waters, this dolphin population has been the focus of annual monitoring program since mid-1990s. However, despite the multi-year efforts^42^,^43^, the current understanding of the population parameters and structure remains severely inadequate, which for well over a decade has led to poorly informed management decisions^31^. Only recently a rigorous collaborative research effort across the PRD and across the administrative border has been initiated by researchers from Hong Kong and mainland China, aiming at providing robust quantitative estimates of the population parameters and demographic processes that determine its biological persistence^31^. This work is currently ongoing. In the meantime, however, an approach that can facilitate quantitative estimates of population viability, if timely, could greatly assist the ongoing research and population monitoring program. Here, we propose such an approach that is both practical and achievable. We explore the threshold survival rate relative to conservation status classification and estimate MVP_k_ for the PRD humpback dolphins. Furthermore, we estimate the threshold habitat size that can hold sufficient number of animals to resist minimal stochasticity, delineating a baseline for a habitat-oriented conservation plan.

## Results

### Threshold of non-calf survival rate to classify the risk of extinction

**Figure 2:**
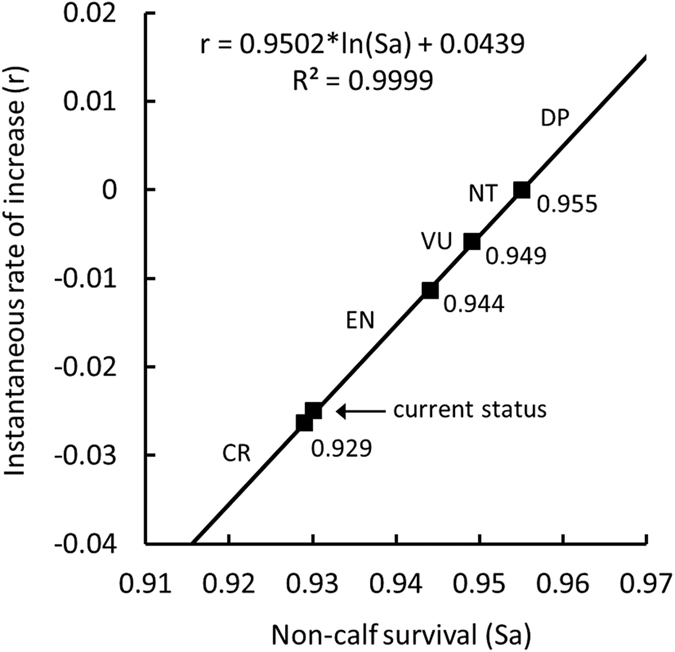
Natural-log relation between instantaneous rate of increase (*r*) and non-calf survival rate (*Sa*) for the Indo-Pacific humpback dolphins in the Pearl River Delta. Cut-off points correspond to threshold *r*_*T*_ (summarized in Table 4) to classify conservation status under the IUCN Criterion A3b^1^. The current population status^8^ is also shown. DP: demographically persistent, NT: Near Threatened, VU: Vulnerable, EN: Endangered, CR: Critically Endangered.

With the application of the least-square method, the projected rate of population change (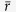) plotted against non-calf survival rate (*Sa*) (Fig. 2), was quantified as


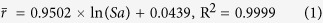


The threshold values of the rate of population change (*r*_T_) representing decline of 30%, 50% and 80% within three generations, which corresponds to the status change from *NT* to *VU, VU* to *EN*, and *EN* to *CR* under the IUCN Criterion A3b^1^ were −0.0058, −0.0113 and −0.0263, respectively (Table 1). Thus, the threshold values of non-calf survival rate representing the rate of population decline corresponding to the status classification as *NT, VU, EN* and *CR* were 0.955 (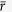 = 0), 0.949 (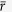 = −0.0058), 0.944 (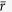 = −0.0113) and 0.929 (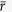 = −0.0263), respectively (Fig. 2). Further, applying this equation to the recent 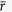
estimate (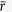 = −0.0249)^8^, the mean *Sa* of the PRD humpback dolphins was estimated at 0.930, which is close to the threshold value that corresponds to the status classification as *CR* (Fig. 2).

### MVP_k_ and MACH estimates

**Figure 3:**
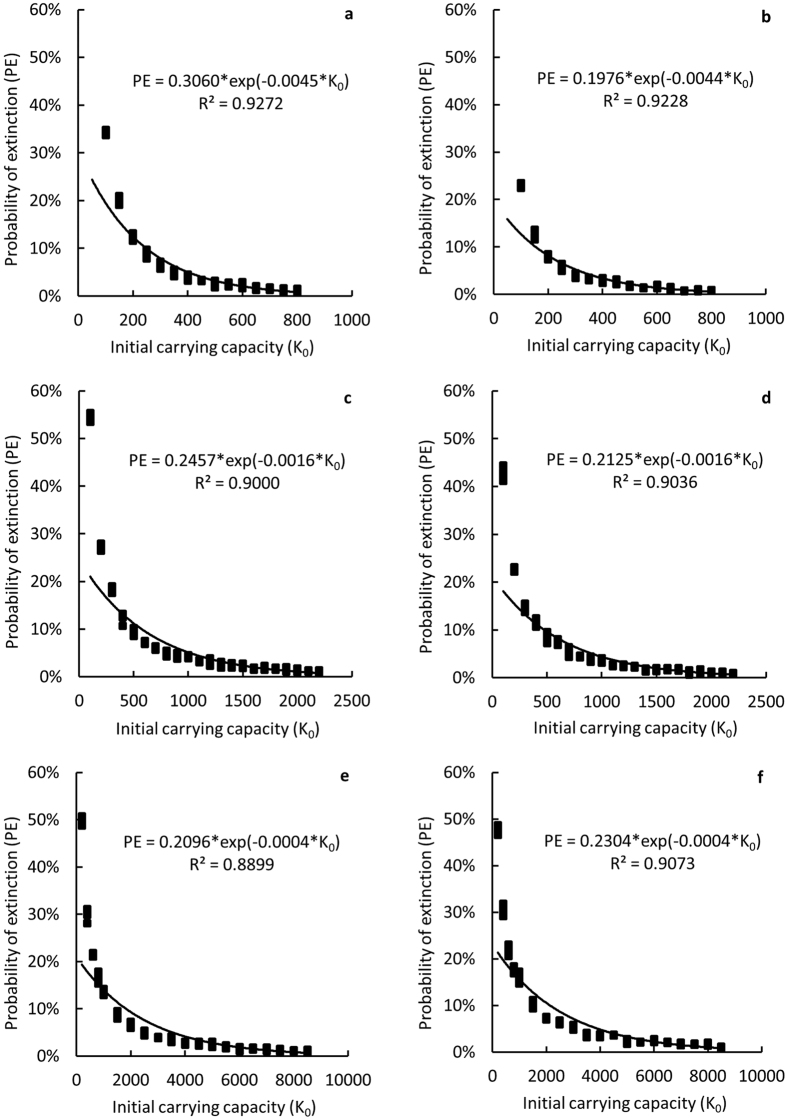
Relation between probabilities of extinction (PE) of humpback dolphins within 40 generations^17^ and habitat carrying capacity (*K*_0_) in the PRD. Scenarios: stationary population (**c,d**), −5% adult mortality (**a,b**), and +5% adult mortality (**e,f**). Models used: VORTEX model (**a,c,e**) and individual-based stage model (IBSM) (**b,d,f**). An exponential regression was fitted with R^2^ ranging from 0.8899 to 0.9272.

The PE (probabilities of extinction) estimates for the PRD humpback dolphins over 40 generations (40 × *T*_0_), where the generation length *T*_0_ = 20.4 years^44^, decreased exponentially with increasing carrying capacity (*K*_0_) for both VORTEX model and individual-based stage matrix (IBSM) model (Fig. 3) in all simulated scenarios. For a stationary population, if PE were to be ≤ 0.01, which was defined as the minimum viable population in carrying capacity (MVP_k_)^45^, the value *K*_0_ had to exceed 2039 or 1932 dolphins according to VORTEX model or IBSM model, respectively (Table 2, column c and d). For a 5% increase and decrease of the adult mortality rate, as compared to the stationary scenario, the MVP_k_ values were projected at 7490–8036 (Table 2, column e and f) or 675–752 (Table 2, column a and b), respectively.

In the PRD, mean density estimates of humpback dolphins approximate 0.690 animals-km^−2^ averaged between wet and dry seasons^46^. Consequently, the minimum area of critical habitat (MACH) for the PRD humpback dolphins was estimated at 2955 km^2^ or 2800 km^2^ based on VORTEX model and IBSM model, respectively (Table 2, column c and d). Under a simulated 5% decrease of the adult mortality rate, MACH estimate was projected at ~1000 km^2^ (Table 2, column a and b), but was almost four-fold that of stationary population when calculated for a simulated 5% increase in the adult mortality rate (Table 2, column e and f) and exceeded the physical size of the PRD when calculated for the current status (Fig. 2) of the PRD population.

## Discussion

Our results indicate that with stationary population structure and suitable environmental conditions, a population of *ca*. 2000 individuals can maintain the persistence of Indo-Pacific humpback dolphins in the Pearl River Delta region across 40 generations (*ca*. 800 years^44^). Although a thorough quantitative assessment of the population figures across the PRD is still lacking, preliminary abundance estimate dating back to 2008 suggests that at the time there were ~2500 humpback dolphins in the PRD waters^46^. Given the current population trend, however, with annual decline rate of ~2.5%^8^, it would have taken ~9 years for the population numbers to drop from 2500 to 2000 individuals, suggesting that at the time of writing this manuscript (December 2016) the humpback dolphin population in the PRD region is fast approaching, or perhaps already at the brink of the MVP_k_ threshold level. Under such circumstances, adverse consequences of demographic stochasticity may likely soon set in and further impair the population viability.

Our model projections indicate that MVP_k_ is highly sensitive to fluctuations in adult survival rate. Even slight changes in the non-calf survival rate of merely 0.225% (or 5% difference in non-calf mortality rate) may considerably affect the MVP_k_ and have major implications on the population trend. Consequently, although a population of *ca*. 2000 individuals can theoretically persist in the PRD across a long timeframe, if so limited it would be resilient to only a minimal risk scenario and remain at a level dangerously close to the risk of stochastic extirpation^47^.

In small populations, besides demographic stochasticity, factors such as environmental stochasticity, genetic deterioration and random catastrophic events can significantly increase the risk of random extinction^17^,^23^,^24^. The numbers needed to resist genetic-diversity loss and maintain evolutionary potential are generally at least 10–20 fold of the number to maintain demographic persistence^17^,^26^,^45^. In our model simulations, we deployed several scenarios, including that of Vulnerable (*VU*) and Endangered (*EN*) under the IUCN criteria and the current population status quantified by Huang *et al*.^8^, each with notably lower non-calf survival rates than that of stationary population (*Sa* = 0.949, 0.944 and 0.930 for *VU, EN* and the current status, respectively; Fig. 2). The resulting estimates were unreasonably high, with *K*_0_ reaching 65,000 under the *VU* scenario if the probability of extinction (PE) was to be maintained ≤0.01. This estimate was over 10 fold larger under the *EN* scenario. For the current population status, with *Sa* = 0.930, which is very close to the *CR* status (*Sa* = 0.929) (Fig. 2), the estimated probabilities of extinction were equal to one (PE = 1) even with *K*_0_ = 1000000, implying that under the current trend the population is deemed to become extinct unless effective conservation measures can rapidly reverse the current population trend.

The MVP_k_ estimate should not be literally interpreted as the minimum number of animals in a population that can withstand stochastic extinction. Instead, it should be seen as baseline for essential habitat size, i.e. the minimum area of critical habitat (MACH) to accommodate a minimum number of animals that can withstand minimal stochasticity^26^,^48^. The MACH estimate may therefore function as a threshold size for an effective protected area design, to accommodate sufficient number of animals that can resist minimal stochastic extinction^13^,^24^,^49^,^50^. In this study, MACH for the PRD humpback dolphins under stationary population structure was estimated at *ca*. 2800~3000 km^2^. This estimate increased four-fold with a 5% increase of the adult mortality rate (Table 2), and exceeded the size of the PRD when calculated for the current status of the PRD population. On the other hand, however, all currently enacted marine protected areas (MPAs) within PRD fail to protect even a bare minimum of the habitat size projected under the most optimistic of the scenarios modelled in our study (Table 3). Furthermore, the largest of the three MPAs in the PRD, the Guangdong Pearl River Estuary Chinese White Dolphin National Nature Reserve, has numerous cargo ships and high-speed ferries routinely passing through its waters, and a large-scale infrastructure under construction which crosses the designated core and buffer zones^31^, undermining the very purpose of the protected area designation. Consequently, a review and reconsideration of the effectiveness of current protected area design in the PRD is urgently needed.

As the primary habitats of humpback dolphins are confined within a narrow band of shallow inshore waters^27^, where the animals depend on limited inshore resources within their already restricted shallow-water distribution^31^,^51^,^52^, they are especially susceptible to habitat degradation and fragmentation^4^. Whether the degradation of habitat is through alteration of near-shore environments or coastal overexploitation^34^,^37^,^38^, or whether it is a more severe case of habitat destruction through urban and/or industrial coastal developments, modification of shorelines and land reclamation^30^,^31^,^53^, it all affects the effective size and structure of dolphin habitats and eventually leads to habitat fragmentation^30^,^34^,^54^. When the effective carrying capacity of each habitat fragment becomes substantially lower than MVP_k_, it lowers the population’s resilience to stochastic and catastrophic events, even if the entire population size may still be larger than MVP_k_.

A recent study by Or^51^ provides an example of a step-wise approach to the identification and prioritisation of key areas and habitats for the conservation of humpback dolphins in Hong Kong and eastern PRD, and a hierarchical two-tier approach to the designation of MPA that may prove effective in long-term conservation. Currently, however, not only the percentage of dolphin core areas under any form of protection is very small, but much of the existing MPAs do not encompass any of the dolphins’ primary habitats^51^. In Hong Kong, for example, <17% of the dolphins’ core areas and <7% of their core foraging grounds are under legal protection^51^, and the majority of the MPAs currently under consideration by Hong Kong authorities include very little of the dolphins’ core foraging grounds^31^,^51^. An identification of core areas used by the dolphins across the PRD and setting up a network of MPAs that offer the necessary legal protection to those key areas and habitats should represent a first step in formulating an effective conservation strategy. Next, enhanced protection measures (e.g. establishment of development-free marine reserves within a larger MPA, with strict regulations of sea traffic and fishing) may provide means of preserving the habitat quality; while protection of areas used by the dolphins to move between their key foraging grounds can secure traveling corridors and afford the functional connectivity within so designated MPAs^31^,^51^, increasing their effectiveness.

The ultimate goal of the conservation efforts across in the PRD should be to increase the survival rate of adults, the primary measure of the population viability. As the survival rate of calves appears to be low in PRD waters^55^, efforts of improving calf survival should also be given high priority as it will lead to a higher future recruitment to the reproductive part of the population. Although this may sounds obvious, the means of achieving this goal are considerably less so; especially in a region that is under a tremendous anthropogenic pressure^31^. Developing effective measures of habitat protection and, first of all, appropriate identification and prioritisation of areas designated for conservation^31^,^51^,^56^ may provide means of reaching the long-term conservation goal. In other words, with reference to the results of our model projections, securing habitat integrity and enhancing the effectiveness of conservation measures which in turn may lead to higher survival rates, can lower the required MACH and bring it to economically affordable and logistically manageable levels.

Given the urgency of the conservation issues facing humpback dolphins in the PRD, our proposed transformation of the IUCN classification criteria of the percentage decline in three generations (30%, 50% or 80% decline; Criterion A3b^1^) into thresholds of non-calf/adult survival rate (Fig. 3) can be applied in the monitoring of population status as part of integrated management program. Estimates of population survival rates can be obtained through either the construction of life tables^8^,^57^, or by means of photo-ID mark-recapture studies^7^,^22^,^58^. The former requires large sample sizes of recovered carcasses, which in the case of cetaceans may take many years to collect. The photo-ID approach, on the other hand, can provide the *Sa* estimates within a reasonable timeframe of 4–5 field seasons and is therefore effective in providing timely assessments of population parameters, status and trend^59^. In cases such as the PRD humpback dolphins, where it is not crisis prevention anymore but crisis management that is at stake, this approach is considerably more effective than the traditional transect survey techniques. Huang *et al*.^8^ argues that it would take a lifespan of 1–3 generations of the PRE humpback dolphins to have the recently estimated trend (~2.5% decline per annum) detected by the line-transect monitoring programme (which currently represents the norm in Hong Kong and the PRD). During that time, a substantial part of the population would have been already lost. A revision of the current monitoring strategy is therefore highly advisable and should be considered as a matter of urgency (see Chan and Karczmarski^58^). Furthermore, we postulate that the approach recommended here (periodic re-assessment of adult survival rate) can be effectively applied to other coastal species elsewhere and prove useful in many cetacean conservation projects.

A recent genetic study investigating the demographic trajectory of the PRD humpback dolphins across several tens of generations^60^ suggests that the contraction of the PRD coastal habitat is among the major historic causes of a gradual population decline that has been ongoing for the past several hundred years, in which time the influence of climate on environmental change has been surpassed by anthropogenic impacts^60^. In recent decades, the process of habitat degradation, fragmentation and loss has vastly accelerated due to major overexploitation, urbanisation and industrialisation of the PRD region^5^,^31^. These recent forms of habitat destruction would not have left yet a detectable genetic signature, but their cumulative impacts are likely far greater than the previous two thousand years of gradual environmental change^60^.

Our findings presented in this report should be seen as a cause for concern and warning sign that the PRD dolphin population barely manages to withstand the current levels of environmental stress. Further environmental degradation (e.g. large-scale habitat loss) is likely to further compromise the population long-term viability, a process that can be expected to accelerate as the population reaches its threshold of demographic stochasticity. The gravity of this issue can hardly be overstated, especially given the ongoing and planned coastal infrastructure projects^31^, and so is the urgency to exercise effective conservation management. To be effective, however, conservation measures should not only increase the volume of the habitat under protection but, importantly, focus the conservation effort on the core areas and key habitats used by the dolphins for their daily needs. Preserving the ecological integrity of those areas should be among the primary conservation targets. Establishing measures preventing further habitat fragmentation along with actions to reconnect fragmented humpback dolphin populations in Chinese coastal waters should be among the top regional conservation priorities.

## Methods

### Thresholds to classify population status

The risk of extinction estimates were classified as *NT, VU, EN* or *CR* under Criterion A3b^1^ using the projected percentage of population decline in the future next three generations. The 30%, 50% and 80% threshold rates of decline within three generations, were respectively applied to define the threshold values of instantaneous rate of increase (*r*_T_) by an exponential model:


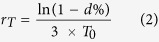


where *d%* is the percentage of decline (i.e. 30%, 50% or 80%) and *T*_0_ is the generation length (*T*_0_ = 20.4 years^44^) of the humpback dolphin.

### Relationship between non-calf survival rate and population change

A hypothetical value of the rate of population change (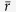) was calculated using a standard method as summarized by Krebs^61^:


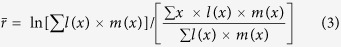


where *l*(*x*) and *m*(*x*) represent age-specific survivorship and reproductive rate at age *x*, respectively. For the humpback dolphin, as for many other cetacean species, precise estimates of the reproductive rate are not readily available; therefore we define *m*(*x*) as follows:


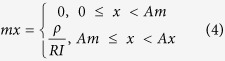


where *RI, Am* and *Ax* represent the calving interval (in years), age at reaching maturity (in years) and life span (in years) of female humpback dolphins, respectively^5^,^8^. The ratio of female offspring per brood, *ρ*, was assumed to be 0.50. A hypothetical age-specific survivorship model, *l*(*x*), was defined using recursive method^16^, similarly as in Huang *et al*.^4^,^8^:


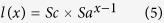


where *x* is the age of animals, *Sc* and *Sa* are the survival rates of calves (*x* < 1) and non-calves (*x* ≥ 1), respectively; the initial value in calculation of *l*(*x*) is 1, i.e. *l*(*0*) = 1^16^,^18^,^57^,^61^. The non-calf survival rate (*Sa*) was defined as a fixed value for all age classes except the calf, which is a common practice in *Sa* estimate from photo-ID studies^7^,^22^.

For the PRD humpback dolphins, the most plausible survival rate of calves (*Sc*) was estimated at 0.61 based on stranding data^55^. To build a relation between the hypothetical value of the rate of population change (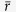) and non-calf survival rates (*Sa*) we calculated 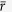
for *Sa* values ranging from 0.90 to 0.98. Survival rates outside this range would be either too low (*Sa* ≤ 0.90) for a population to viably persist, or overly optimistic (*Sa* ≥ 0.98) for the humpback dolphin^4^. The calculation of 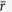
under each *Sa* was repeated by 5,000 iterations that adopted *Am, RI* and *Ax* re-sampled from within their known range (Table 4) to factor in the effect of parameter uncertainty^4^,^5^,^8^. A non-linear regression using natural-log function (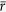 = *a* × ln(*Sa*) + b) was applied to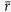, which was further used to determine the threshold *Sa* values corresponding to the threshold values of instantaneous rate of increase (*r*_*T*_).

### MVP in carrying capacity (MVP_k_) and minimum area of critical habitat (MACH)

The minimum viable population in carrying capacity (MVP_k_) was defined as the threshold value of the carrying capacity (*K*_0_) that facilitates the population persistence over at least 40 generations with ≤0.01 probability of extinction (PE)^45^. The PE was projected with population viability analysis (PVA) using VORTEX model^62^ and an individual-based stage matrix (IBSM) model (see Huang and Karczmarski^5^ and Huang *et al*.^8^ for details) specifically designed to fit the reproductive parameters of the PRD humpback dolphins (Table 4)^5^,^8^. In VORTEX model we excluded the influence from inbreeding depression and environmental catastrophes as neither quantitative nor qualitative baseline of these two factors are available for the PRD humpback dolphins. Parameters used to run VORTEX are summarized in the Table 5. The initial abundance (*N*_0_) was defined at *K*_0_. For both models, the value of *Sa* was initially defined at
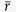
= 0, i.e. only stochastic, not demographic factors influence the population change. Subsequently, simulations with a 5% increase and decrease in adult mortality rates, as compared to the stationary scenario, were also run to test the sensitivity of MVP_k_ to the change of non-calf survival rate. We applied the PE estimates to a model as follows:


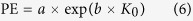


where coefficients *a* and *b* are estimated by least-square method. MVP_k_ was determined as the value of *K*_0_ when PE = 0.01^45^.The minimum area of critical habitat (MACH) estimate was calculated using the following formula^24^,^26^:


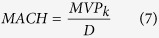


where *D* is the population density estimate from Chen *et al*.^46^.

## Additional Information

**How to cite this article**: Karczmarski, L. *et al*. Threshold of long-term survival of a coastal delphinid in anthropogenically degraded environment: Indo-Pacific humpback dolphins in Pearl River Delta. *Sci. Rep.* **7**, 42900; doi: 10.1038/srep42900 (2017).

**Publisher's note:** Springer Nature remains neutral with regard to jurisdictional claims in published maps and institutional affiliations.

## Figures and Tables

**Figure 1:**
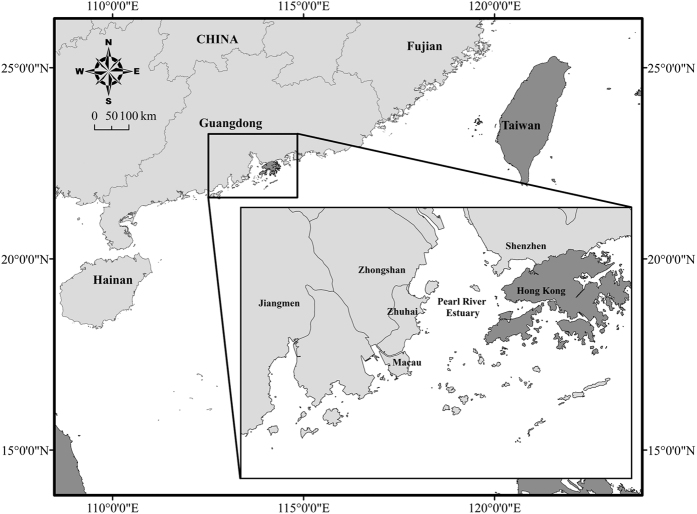
The Pearl River Delta (PRD) region on the south-east coast of China. This low-lying subtropical coastal region that surrounds the Pearl River Estuary (PRE) is among the most industrialised and densely urbanised regions in the world. The map was generated using software ArcGIS (Version 10.2, http://www.esri.com/software/arcgis).

**Table 1:** Threshold values of % decline and instantaneous rate of increase (*r*_*T*_) for the Indo-Pacific humpback dolphin in Pearl River Delta applied for the status classification under Criterion A3b of IUCN Red List Categories and Criteria^1^.

Status	% decline	*r*_*T*_
NT	0–30%	−0.0058 ~ 0
VU	30–50%	−0.0113 ~ −0.0058
EN	50–80%	−0.0263 ~ −0.0113
CR	≥80%	≤−0.0263

**Table 2:** Minimum viable population in carrying capacity (MVP_k_) and minimum area of critical habitat (MACH) estimates for the PRD humpback dolphins.

		−5% mortality		Stationary		+5% mortality	
		(a) VORTEX	(b) IBSM	(c) VORTEX	(d) IBSM	(e) VORTEX	(f) IBSM
MVP_k_	Estimate	752	675	2039	1932	7490	8036
	95% C.I.	729–777	655–697	1973–2112	1872–1998	7191–7825	7736–8370
MACH# (km^2^)	Estimate	1089.9	978.3	2955.1	2800.0	10855.1	11646.4
	Range	1056.5–1126.1	949.3–1010.1	2859.4–3060.9	2713.0–2895.7	10421.7–11340.6	11211.6–12130.4

Scenarios: stationary population (c,d), −5% adult mortality (a,b) and +5% adult mortality (e,f). Models used: VORTEX model (a,c,e) and individual-based stage model (IBSM) (b,d,f).

^#^MACH was calculated as 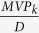 where *D* is the population density estimate from Chen *et al*.^46^ that averages 0.690 animals-km^−2^ across all survey areas in the Pearl River Delta region and in both ‘wet’ and ‘dry’ seasons.

**Table 3:** Current Marine Protected Areas (MPAs) specifically designated for the conservation of Indo-Pacific humpback dolphins in the PRD and the cumulative size of the estuarine habitat under legal protection.

MPA	Area (km^2^)
Guangdong Pearl River Estuary Chinese White Dolphin National Nature Reserve (mainland China waters)	460
Guangdong Jiangmen (Taishan Daijin Island) Chinese White Dolphin Provincial Nature Reserve (mainland China waters)	108
Sha Chau and Lung Kwu Chau Marine Park (Hong Kong waters)	12
Total	580

**Table 4:** The range of the age at reaching sexual maturity, referred here as age at maturity (*Am*), reproductive interval (*RI*) and expected lifespan (*Ax*) of female humpback dolphins in Pearl River Delta.

Parameters	Lower	Upper	References
*Am* (years)	9	11	Jefferson *et al*.^55^, Jefferson^42^
*RI* (years)	2	5	Jefferson *et al*.^55^, Jefferson^42^
*Ax* (years)	38	43	Huang *et al*.^8^, Jefferson *et al*.^55^, Jefferson^42^

**Table 5:** Parameterization for VORTEX model used to determine the probabilities of extinction within 40 generations^17^ of humpback dolphins, per generation length of 20.4 years^44^.

Parameters	Values
number of iterations	2000
number of years	816
reproductive system	polygynous
age of first offspring for females	9
age of first offspring for males	11
maximum age of reproduction	38
maximum number of broods per year	1
maximum number of progeny per brood	1
sex ratio at birth - in % males	50%
% males in breeding pool	100%
reproductive rates	28.57%
environmental variation in % breeding	30%
initial population size (*N*_0_)	=initial carrying capacity (*K*_0_)
